# Supplementary material for: Community perspectives: An exploration of potential barriers to men’s involvement in maternity care in a central Tanzanian community
Source: PLoS One. 2020 May 21;15(5):e0232939. doi: 10.1371/journal.pone.0232939 (PMC7241761; doi:10.1371/journal.pone.0232939)
Supplement: S1 Appendix — (PDF) [file pone.0232939.s001.pdf]

FOCUS GROUP DISCUSSION GUIDE – ENGLISH VERSION

Consent form (Men's focus group discussion)

Title of the study: Community perspectives: an exploration of potential barriers to men's involvement in maternity care in a central Tanzanian community

Introduction –Welcome
We are researchers from the University of Dodoma, College of Health Sciences. We are carrying out a study on perspectives of community towards male involvement in maternal health care. We want to know the barriers of men's involvement in maternity care during pregnancy, delivery and after delivery, so as to improve the overall health of women, children and men in the region.

The information obtained from this study will be used in planning and designing intervention to encourage male involvement in maternal health care. We are requesting you to participate in this research by giving us the information that we need. You are under no obligation to participate. You have the right to withdraw at any time without the need to justify your decision. But we will appreciate greatly if you take part to the end to make the study successful. The information collected from you will be coded so that they are not linked to your name and your views will not be shared with any other person without your permission.

This consent form has been read and explained to me and I voluntarily consent to participate in this study.

…………………………………. ……………………………………
Witness's signature Participant's signature
Age………Education level……………..occupation……………..
(Research Assistant) (Thumb print)
Research………………………………………. Date…………………………………

Focus Group Guide for Men

Name of Village____________ Ward name ___________Ward number_______
Date of discussion ________________Number of participants_____________
Place of usual residence (1) Urban (2) Rural
Name FGD chair__________ other research assistant_____________

Introduction of moderators and observers
We are going to talk about topics related to maternal health care. The aim of this discussion is for you to share your ideas and experiences with us so that we can understand your views that will help in improving the maternal health services for women as well as increase male involvement in maternal health care services, which can result in improvement of the health of family and lead to reduction of maternal and child mortality in the region.

There is no right or wrong answer to the questions that we will be asking you. Please feel free to answer exactly as you feel and respect others' views. Anything you say here will be kept confidential. We will never mention your name outside this room. If you do not want to answer particular questions that is okay. If you need to leave at any time, that is fine too. I request you to allow us tape-record the proceedings and note taking of this meeting.

Introduction of particulars (name, work, etc) -Interviewer

1. **What do you know about ANC, delivery and postnatal care services?**

Probe: Who is the intended user?
Who should attend?
How many times they should attend?
What are the services offered?

2. **Do you think it is important for your partner/husband to attend ANC, delivery and postnatal care services?**

Probe: Any benefits to your social/psychological wellbeing during pregnancy and delivery?
What services directly benefit men?
Would their involvement affect ANC, delivery and postnatal care?

3. **Do men in this village/community accompany their wives/partners for ANC, delivery and postnatal care?**

Probe: What are the benefits of men attending these with their spouses?
To the mother?
To an unborn baby and the newborn?
To the father?

4. **What could be the reasons that prevent men from accompanying their wives/partners to ANC, delivery and postnatal care?**

Probe: Cultural issues? Any social economic issue? Information issues?
Health care unit related factors?
Knowledge gaps of what is done at the health facilities?
Lack of information? Marriage issues

5. **Do men in this village/community share household workload with their partners during pregnancy, delivery and after delivery**

Probe: provide rest to their partners? Washing dishes? Cooking? Child care? Fetching water? Farm work? Washing clothes? Cleaning the house?

Thank you very much for your time and information

Do you have any questions or comments on the above issues we have been discussing?

(Answer any questions raised and thank the participants before closure of the session)

Consent form (Women's focus group discussion)

Title of the study: Community perspectives: an exploration of potential barriers to men's involvement in maternity care in a central Tanzanian community

Introduction –Welcome
We are researchers from University of Dodoma, College of Health Sciences. We are carrying out a study on perspectives of community towards male involvement in maternal health care. We want to know the barriers of men's involvement in maternity care during pregnancy, delivery and after delivery, so as to improve the overall health of women, children and men in the region. The information obtained from this study will be used in planning and designing intervention to encourage male involvement in maternal health care. We are requesting you to participate in this research by giving us the information that we need. You are under no obligation to participate. You have the right to withdraw at any time without the need to justify your decision. But we will appreciate greatly if you take part to the end to make the study successful.

The information collected from you will be coded so that they are not linked to your name and your views will not be shared with any other person without your permission. This consent form has been read and explained to me and I voluntarily consent to participate in this study.

…………………………………. ……………………………………
Witness's signature Participants signature
Age………Education level……………..occupation……………..

(Research Assistant) (Thumb print)
Research………………………………………. Date……………………..

Focus Group Guide for Women

Name of Village____________ Ward name ___________Ward number_______
Date of discussion ________________Number of participants______________
Place of usual residence (1) Urban (2) Rural
Name FGD chair__________ other research assistant_____________

Introduction of moderators and observers
We are going to talk about topics related to maternal health care. The aim of this discussion is for you to share your ideas and experiences with us so that we can understand your views that will help in improving maternal health services for women as well as to increase male involvement in maternal health care services, which can result in improvement of health of the family and lead to reduction of maternal and child mortality in the region.

There is no right or wrong answers to the questions that we will be asking you. Please feel free to answer exactly as you feel and respect for others' views. Anything you say here will be kept confidential. We will never mention your name outside this room. If you do not want to answer

particular questions, that is okay. If you need to leave at any time, that is also fine. I request you to allow us tape the proceedings/note taking of this meeting.

Introduction of particulars (name, work, etc) -Interviewer

1. **What do you know about ANC, delivery and postnatal care services?**

Probe: Who is the intended user?
Who should attend?
What are the services offered?

2. **Do you think it is important for your partner/husband to attend ANC, delivery and postnatal care services?**

Probe: Any benefits to your social/psychological wellbeing during pregnancy and delivery?
What services directly benefit men?
Would their involvement affect ANC, delivery and postnatal care?

3. **Do men in this village/community accompany their wives/partners for ANC, delivery and postnatal care services?**

Probe: What are the benefits of men attending these maternal services with their spouses?
To the mother?
To unborn baby and the newborn? To the father?

4. **What could be the reasons that prevent men to accompany the wives/partners for ANC, delivery and postnatal care services?**

Probe: Culture issues? Any social economic issue?
Health unit related factors?
Knowledge gaps of what is done at the health facilities?
Lack of information? Marriage issues?

5. **Do men in this village/community share household workload with their partners during pregnancy, delivery and after delivery**

Probe: provide rest to their partners? Washing dishes? Cooking? Child care? Fetching water? Farm work? Washing clothes? Cleaning the house?

Thank you very much for your time and information

MWONGOZO WA MAJADILIANO

IDHINI YA MDODOSWAJI (DODOSO LA WANAUME)

Kichwa cha Habari: Mtazamo wa jamii kuhusu mambo yanayowazuia wanaume kujihusisha katika uangalizi wa afya ya uzazi kwa wakina mama katika Jamii ya Tanzania mkoani Dodoma.

Utangulizi

Sisi ni watafiti kutoka chuo kikuu cha Dodoma, tunafanya utafiti kuhusu ushiriki wa wanaume katika uangalizi wa afya ya mama wakati wa kipindi cha uzazi mkoani Dodoma. Utafiti huu unalenga kujua mambo muhimu yanayowazuia wanaume kujihusisha katika uangalizi wa afya ya uzazi kwa akina mama katika kipindi cha uzazi, kama vile kuwasindikiza wenzi wao kwenye huduma za afya wakati wa ujauzito, kujifungua na baada ya kujifungua na pia kuwasaidia kazi za nyumbani.

Maelezo yatakayopatikana katika utafiti huu yatawasaidia wanaohusika na kupanga mipango inayohusu ushiriki wa wanaume katika uangalizi wa afya ya akina mama katika kipindi cha uzazi, kujua mambo muhimu ya kuzingatia wakati wa kupanga mikakati hiyo. Lengo la kupanga mikakati mizuri ni kusaidia kuongeza ushiriki wa wanaume ili kuimarisha afya ya mama, mtoto na baba katika familia na hivyo kupunguza idadi ya vifo vya mama na mtoto vinavyo sababishwa na matatizo ya uzazi katika mkoa kwa ujumla.Tunawaomba mshiriki katika utafiti huu kwa kutueleza yale tunayoyahitaji.

Ushiriki wako katika utafiti huu ni hiari. Una uhuru wa kutoshiriki wakati wowote bila kutoa sababu kuhusu uamuzi wako wa kutoshiriki. Lakini tunawasihi kushiriki hadi mwisho wa majadiliano ili kufanikisha utafiti huu. Maelezo mtakayotoa yatapewa namba ambayo haitahusiana na jina lako, na maoni ya mtu hayata shirikishwa mtu mwingine bila ruhusa yake.

Nimesomewa na kuelezwa maelezo hayo hapo juu, ninakubali kwa hiari yangu kushiriki katika utafiti huu.

………………………………….　　　　　　……………………………………

Sahihi ya shahidi　　　　　　　　　　**Sahihi ya mshiriki**

Sahihi ya mtafiti msaidizi......................................

Sahihi ya mtafiti……………………………………… **Date……..............................**

MWONGOZO WA MAJADILIANO KWA WANAUME

Kichwa cha Habari: Mtazamo wa jamii kuhusu mambo yanayowazuia wanaume kujihusisha katika uangalizi wa afya ya uzazi kwa wakina mama katika Jamii ya Tanzania mkoani Dodoma.

Jina la Kijiji____________ Jina la Kata ____________Namba ya kata______
Jina la Wilaya____________________(1) Mjini (2) Kijijini
Tarehe ya Majadiliano______________Namba ya Group_______Idadi ya washiriki______
Jina la M/kiti wa majadiliano_________________ Watafiti wasaidizi________________
________________________ _______________________ ______________________

Utambulisho wa Mwenyekiti wa Majadiliano na Watafiti Wasaidizi

Majadiliano yetu yatalenga uangalizi wa afya ya mama wakati wa kipindi cha uzazi. Nia ya majadiliano haya ni kujua mtazamo wenu na uzoefu wenu kuhusu mambo yanayo wazuia wanaume kushiriki katika uangalizi wa afya ya mama wakati wa kipindi cha ujauzito, kujifungua na baada ya kujifunguaili. Maoni yenu, yatasaidia kuimarisha huduma ya afya kwa akina mama na kuongeza ushiriki wa wanaume katika huduma ya uangalizi wa afya ya mwanamke katika kipindi cha uzazi. Hii itasaidia kuimarisha afya ya wanawake na watoto na kusaidia kupunguza vifo vya akina mama na watoto katika Mkoa.

Hakuna jibu sahihi au lisilosahihi kwa maswali tutakayokuwa tunauliza. Tafadhali jisikie huru kujibu vile unavyojisikia kujibu. Tuheshimu mawazo ya kila mshiriki. Kila utakachokisema hapa ni siri. Hatutataja jina lako nje ya chumba hiki/mahali hapa. Kama hutaki kujibu swali fulani hiyo ni sawa, kama unataka kuondoka wakati wowote hiyo ni sawa. Ila ninawasii sana tushiriki hadi mwisho wa majadiliano haya kwani mawazo yenu niya msingi sana katika utafiti huu.

Ninawaomba mturuhusu kurekodi/kuandika majadiliano ya mkutano.

Kujitambulisha (umri, elimu, kazi)

6. **Unaelewa nini kuhusu kliniki ya mama mjazito, huduma ya kujifungua na huduma baada ya kujifungua?**

Dodosa: Nani wanalengwa kutumia huduma hii?
Nani wanatakiwa kuhudhuria katika huduma hii?
Ni mara ngapi wanatakiwa wahudhurie?
Ni huduma gani zinatolewa huko?

7. **Je unafikiri ni muhimu wewe kwenda na mwenzi/mke wako katika kliniki kupata huduma za afya wakati akiwa mjamzito, wakati wa kujifungua na baada ya kujifungua?**

Probe: Kunafaida yoyote kwako, kiafya, mawazo hasa wakati wa ujauzito na kujifungua?
Ni huduma gani zinamnufaisha mwanaume?
Ushiriki wa mwanaume katika huduma hii utaleta mabadiliko yoyote katika uangalizi wa afya za akina mama wakati wa ujauzito, kujifungua na baada ya kujifungua?

8. **Je wanaume katika kijiji hiki wanawasindikikiza wake/wenzi wao katika kliniki ya wajawazito, kujifungua na kliniki baada ya kujifungua?**

Dodosa: Ni faida gani wanapata wanaume wakihudhuria kliniki pamoja na wenzi/wake zao?
Mama anapata faida gani?
Mtoto aliyetumboni na mtoto aliyezaliwa anapata faida gani?
Baba wa mtoto anapata faida gani?

9. **Ni sababu zipi zinawazuia wanaume kwenda klininiki na wake/wenza wao wakati wa ujauzito, kujifungua na baada ya kujifungua?**

Dodosa: Mila na desturi? Hali ya kiuchumi? Taarifa kuhusu kuhudhuria kliniki?
Masuala yanayohusiana na kituo cha huduma za afya?
Kukosa uelewa kuhusu kinachofanyika katika kituo cha kutolea huduma?
Kutokuwa na taarifa ? Mambo ya ndoa?

10. **Je wanaume katika kijiji/jamii hii wanawasaidia wenzi/wake zao kazi za nyumbani katika kipindi cha ujauzito, kujifungua na baada ya kujifungua?**

Probe: Wanawapumzisha wenzi wao? Wanaosha vyombo? wanapika? Wanalea watoto? Wanachota maji? Wanaenda shamba? Wanafua nguo? Wanasafisha nyumba?

Asante sana kwa muda wenu na maelezo mliyonipa.
Unaswali lolote au maoni kuhusu haya tuliyojadili?

(Jibu maswali yatakayoulizwa na washukuru washiriki kabla ya kufunga kipindi)

IDHINI YA MDODOSWAJI (DODOSO LA WANAWAKE)

Kichwa cha Habari: Mtazamo wa jamii kuhusu mambo yanayowazuia wanaume kujihusisha katika uangalizi wa afya ya uzazi kwa wakina mama katika Jamii ya Tanzania mkoani Dodoma.

Utangulizi

Sisi ni watafiti kutoka chuo kikuu cha Dodoma, tunafanya utafiti kuhusu ushiriki wa wanaume katika uangalizi wa afya ya mama wakati wa kipindi cha uzazi mkoani Dodoma. Utafiti huu unalenga kujua mambo muhimu yanayowazuia wanaume kujihusisha katika uangalizi wa afya ya uzazi kwa akina mama katika kipindi cha uzazi, kama vile kuwasindikiza wenzi wao kwenye huduma za afya wakati wa ujauzito, kujifungua na baada ya kujifungua na pia kuwasaidia kazi za nyumbani.

Maelezo yatakayopatikana katika utafiti huu yatawasaidia wanaohusika na kupanga mipango inayohusu ushiriki wa wanaume katika uangalizi wa afya ya akina mama katika kipindi cha uzazi, kujua mambo muhimu ya kuzingatia wakati wa kupanga mikakati hiyo. Lengo la kupanga mikakati mizuri ni kusaidia kuongeza ushiriki wa wanaume ili kuimarisha afya ya mama, mtoto na baba katika familia na hivyo kupunguza idadi ya vifo vya mama na mtoto vinavyo sababishwa na matatizo ya uzazi katika mkoa kwa ujumla.Tunawaomba mshiriki katika utafiti huu kwa kutueleza yale tunayoyahitaji.

Ushiriki wako katika utafiti huu ni hiari. Una uhuru wa kutoshiriki wakati wowote bila kutoa sababu kuhusu uamuzi wako wa kutoshiriki. Lakini tunawasihi kushiriki hadi mwisho wa majadiliano ili kufanikisha utafiti huu. Maelezo mtakayotoa yatapewa namba ambayo haitahusiana na jina lako, na maoni ya mtu hayata shirikishwa mtu mwingine bila ruhusa yake.

Nimesomewa na kuelezwa maelezo hayo hapo juu, ninakubali kwa hiari yangu kushiriki katika utafiti huu.

………………………………….　　　　　　……………………………………

Sahihi ya shahidi　　　　　　　　　　**Sahihi ya mshiriki**

Sahihi ya mtafiti msaidizi.......................................

Sahihi ya mtafiti……………………………………… **Date……..............................**

MWONGOZO WA MAJADILIANO KWA WANAWAKE

Kichwa cha Habari: Mtazamo wa jamii kuhusu mambo yanayowazuia wanaume kujihusisha katika uangalizi wa afya ya uzazi kwa wakina mama katika Jamii ya Tanzania mkoani Dodoma.

Jina la Kijiji____________ Jina la Kata ____________Namba ya kata______
Jina la Wilaya____________________(1) Mjini (2) Kijijini
Tarehe ya Majadiliano____________Namba ya Group______Idadi ya washiriki_____
Jina la M/kiti wa majadiliano_________________ Watafiti wasaidizi________________
________________________ _______________________ ______________________

Utambulisho wa Mwenyekiti wa Majadiliano na Watafiti Wasaidizi

Majadiliano yetu yatalenga uangalizi wa afya ya mama wakati wa kipindi cha uzazi. Nia ya majadiliano haya ni kujua mtazamo wenu na uzoefu wenu kuhusu mambo yanayo wazuia wanaume kushiriki katika uangalizi wa afya ya mama wakati wa kipindi cha ujauzito, kujifungua na baada ya kujifunguaili. Maoni yenu, yatasaidia kuimarisha huduma ya afya kwa akina mama na kuongeza ushiriki wa wanaume katika huduma ya uangalizi wa afya ya mwanamke katika kipindi cha uzazi. Hii itasaidia kuimarisha afya ya wanawake na watoto na kusaidia kupunguza vifo vya akina mama na watoto katika Mkoa.

Hakuna jibu sahihi au lisilosahihi kwa maswali tutakayokuwa tunauliza. Tafadhali jisikie huru kujibu vile unavyojisikia kujibu. Tuheshimu mawazo ya kila mshiriki. Kila utakachokisema hapa ni siri. Hatutataja jina lako nje ya chumba hiki/mahali hapa. Kama hutaki kujibu swali fulani hiyo ni sawa, kama unataka kuondoka wakati wowote hiyo ni sawa. Ila ninawasii sana tushiriki hadi mwisho wa majadiliano haya kwani mawazo yenu niya msingi sana katika utafiti huu.

Ninawaomba mturuhusu kurekodi/kuandika majadiliano ya mkutano.

Kujitambulisha (umri, elimu, kazi)

1. **Unaelewa nini kuhusu kliniki ya mama mjazito, huduma ya kujifungua na huduma baada ya kujifungua?**

Dodosa: Nani wanalengwa kutumia huduma hii?
Nani wanatakiwa kuhudhuria katika huduma hii?
Ni mara ngapi wanatakiwa wahudhurie?
Ni huduma gani zinatolewa huko?

2. **Je unafikiri ni muhimu mumeo/mwenzi wako kwenda na wewe kliniki kupata huduma za afya ukiwa mjamzito, wakati wa kujifungua na baada ya kujifungua?**

Dodosa: Kunafaida yoyote kwako, kiafya, mawazo hasa wakati wa ujauzito na kujifungua?
Ni huduma gani zinamnufaisha mwanaume?
Ushiriki wa wanaume katika huduma hii utaleta mabadiliko yoyote katika uangalizi wa afya za akina mama wakati wa ujauzito, kujifungua na baada ya kujifungua?

3. **Je wanaume katika kijiji hiki/jamii hii wanawasindikikiza wake/wenzi wao katika kliniki ya wajawazito, kujifungua na kliniki baada ya kujifungua?**

Dodosa: Ni faida gani wanapata wanaume wakihudhuria kliniki pamoja na wenzi/wake zao?
Mama anapata faida gani?
Mtoto aliyetumboni na mtoto aliyezaliwa anapata faida gani?
Baba wa mtoto anapata faida gani?

4. **Ni sababu zipi zinawazuia wanaume kwenda klininiki na wake/wenza wao wakati wa ujauzito, kujifungua na baada ya kujifungua?**

Dodosa: Mila na desturi? Hali ya kiuchumi? Taarifa kuhusu kuhudhuria kliniki?
Masuala yanayohusiana na kituo cha huduma za afya?
Kukosa uelewa kuhusu kinachofanyika katika kituo cha kutolea huduma?
Kutokuwa na taarifa? Mambo ya ndoa?

5. **Je wanaume katika kijiji/jamii hii wanawasaidia wenzi wao/wake zao kazi za nyumbani katika kipindi cha ujauzito, kujifungua na baada ya kujifungua?**

Dodosa:Wanawapumzisha wenzi wao? Wanaosha vyombo? wanapika? Wanalea watoto? Wanachota maji? Wanaenda shamba? Wanafua nguo? Wanasafisha nyumba?

Asante sana kwa muda wenu na maelezo mliyonipa.

Unaswali lolote au maoni kuhusu haya tuliyojadili?

(Jibu maswali yatakayoulizwa na washukuru washiriki kabla ya kufunga kipindi)
