# Supplementary material for: Community perspectives: An exploration of potential barriers to men’s involvement in maternity care in a central Tanzanian community
Source: PLoS One. 2020 May 21;15(5):e0232939. doi: 10.1371/journal.pone.0232939 (PMC7241761; doi:10.1371/journal.pone.0232939)
Supplement: S2 Appendix — (PDF) [file pone.0232939.s002.pdf]

INTERVIEW GUIDE – ENGLISH VERSION

Consent form (Key Informant)

Title of the study: Community perspectives: an exploration of potential barriers to men's involvement in maternity care in a central Tanzanian community

Introduction –Welcome
We are researchers from University of Dodoma, College of Health Sciences. We are carrying out a study on perspectives of community towards male involvement in maternal health care. We want to know the barriers of men's involvement in maternity care during pregnancy, delivery and after delivery, so as to improve the overall health of women, children and men in the region. The information obtained from this study will be used in planning and designing intervention to encourage male involvement in maternal health care. We are requesting you to participate in this research by giving us the information that we need. You are under no obligation to participate. You have the right to withdraw at any time without the need to justify your decision. But we will appreciate greatly if you take part to the end to make the study successful.

The information collected from you will be coded so that they are not linked to your name and your views will not be shared with any other person without your permission.
This consent form has been read and explained to me and I voluntarily consent to participate in this study.

………………………………….	……………………………………
Witness's signature	Participants signature

Age………Education level……………..occupation……………..

(Research Assistant) (Thumb print)
Research………………………………………. Date………………….

Name of Village____________ Ward name ____________Ward number_______
Date of discussion____________________ Name of interviewer____________________
Place of usual residence (1) Urban (2) Rural

Key Informant Guide

Introduction of moderators and observers
We are going to talk about topics related to maternal health care. The aim of this discussion is for you to share your ideas and experiences with us so that we can understand your views that will help in improving the maternal health services for women as well as increase male involvement in maternal health care services, which can result in improvement of family health and lead to reduction of maternal and child mortality in the region.

There is no right or wrong answers to the questions that we will be asking you. Please feel free to answer exactly as you feel. Anything you say here will be kept confidential. We will never mention your name outside this room. If you do not want to answer particular questions it is fine. I request you to allow me to tape-record the proceedings/note taking of this interview.

Introduction of particulars (name, work, etc) - Interviewer

1. **What is your opinion about the level of men's accompanying their wives/partners to ANC, delivery and postnatal care services?**

2. **Do you think it is important for the husbands/partners to discuss with their wives and attend ANC, delivery and postnatal care?**

Probes: What are the benefits of this action to the woman, unborn baby and the man?
How does male involvement improve the quality ANC, delivery and postnatal care and utilization of these services by pregnant women and mothers?
Would their involvement affect ANC, delivery and postnatal care services?

3. **Do men in this area accompany their wives/partners to ANC, delivery and postnatal care?**

Probes: What are the benefits of men attending these services with their spouses?
To the mother?
To unborn baby and the newborn?
To the father?

4. **What are the possible reasons that could prevent men from accompanying their wives on ANC, delivery and postnatal care?**

Probes: Any health facility issues?
Any culture issues?
Any policy issues?
Any social economic issues?
Any information gap?

5. **Do men in this village/community share household workload with their partners during pregnancy, delivery and after delivery**

Probe: provide rest to their partners? Washing dishes? Cooking? Child care? Fetching water? Farm work? Washing clothes? Cleaning the house?

Do you have any questions or comments on the above issues we have been discussing?

Thank you very much for your time and information

IDHINI YA MDODOSWAJI (DODOSO LA WATU MAALUM)

Kichwa cha Habari: Mtazamo wa jamii kuhusu mambo yanayowazuia wanaume kujihusisha katika uangalizi wa afya ya uzazi kwa wakina mama katika Jamii ya Tanzania mkoani Dodoma.

Sisi ni watafiti kutoka chuo kikuu cha Dodoma. Tunafanya utafiti kuhusu ushiriki wa wanaume katika afya ya uzazi Mkoani Dodoma. Utafiti huu unalenga kujua mambo muhimu yanayowazuia wanaume kujihusisha na uangalizi wa Afya ya uzazi kwa wakina mama, na pia kujua ni kwanini wanaume wengi katika Mkoa huu hawajihusishi na uangalizi wa afya za wenzi wao wakati wa ujauzito, kama vile kuwasindikiza wenzi wao kwenye huduma za afya wakati wa ujauzito, kujifungua na baada ya kujifungua na pia kuwasaidia kazi.

Utafiti huu hauta kunufaisha wewe moja kwa moja, ila utasaidia kupata maelekezo ambayo yatawasaidia wale wanohusika na kupanga mipango kuhusu huduma ya afya ya uzazi kujua ni mambo gani ya kuzingatia katika mipango yao ili kuwafanya wanaume wengi kutumia huduma hii ya afya ya uzazi, na kuwafanya wajihusishe na uangalizi wa afya za wenzi wao wakati wa ujauzito, kujifungua na baada ya kujifungua. Hii itasaidia kuimarisha afya ya baba, mama na mtoto katika familia. Hivyo kupunguza vifo vya mama na mtoto vinavyosababishwa na matatizo ya uzazi

Utafiti huu unatambuliwa na umeruhusiwa na mamlaka zinazohusika. Utafiti huu utatumia njia ya mahojiano. Kwako wewe unachotakiwa kufanya ni kujibu maswali utakayoulizwa. Mahojiano yatakuchua muda wa takribani nusu saa. Una uhuru wa kuuliza swali lolote, wakati wowote kuhusiana na utafiti huu iwapo utahitaji maelezo zaidi.

Ushiriki wako katika utafiti huu ni hiari. Una uhuru wa kutoshiriki wakati wowote bila kutoa sababu kuhusu uamuzi wako wa kutoshiriki. Maelezo utakayotoa yatapewa namba ambayo haitahusiana na jina lako na maoni yako hayata shirikishwa mtu mwingine bila ruhusa yako.

Nimesoma/Nimeelezwa maelezo hayo hapo juu, ninakubali kwa hiari yangu kushiriki katika utafiti huu.

Sahihi ya mdodoswaji___________________ Tarehe________________

Nimemweleza na kumwelewesha mshiriki hapo juu kuhusu utafiti huu na ameidhinisha ushiriki wake.

Sahihi ya mdodosaji_________________ Tarehe________________

MWONGOZO WA MAJADILIANO KWA WATU MAALUM

Kichwa cha Habari: Mtazamo wa jamii kuhusu mambo yanayowazuia wanaume kujihusisha katika uangalizi wa afya ya uzazi kwa wakina mama katika Jamii ya Tanzania mkoani Dodoma.

Jina la Kijiji____________ Jina la Kata ____________Namba ya kata______
Jina la Wilaya____________________(1) Mjini (2) Kijijini
Tarehe ya mahojiano____________________Namba ya Mdodoswaji___________________
Jina la Mdodosaji_________________________________

Utambulisho wa Mwenyekiti wa Majadiliano na Watafiti Wasaidizi

Majadiliano yetu yatalenga uangalizi wa afya ya mama wakati wa kipindi cha uzazi. Nia ya majadiliano haya ni kujua mtazamo wenu na uzoefu wenu kuhusu mambo yanayo wazuia wanaume kushiriki katika uangalizi wa afya ya mama wakati wa kipindi cha ujauzito, kujifungua na baada ya kujifunguaili. Maoni yenu, yatasaidia kuimarisha huduma ya afya kwa akina mama na kuongeza ushiriki wa wanaume katika huduma ya uangalizi wa afya ya mwanamke katika kipindi cha uzazi. Hii itasaidia kuimarisha afya ya wanawake na watoto na kusaidia kupunguza vifo vya akina mama na watoto katika Mkoa.

Hakuna jibu sahihi au lisilosahihi kwa maswali tutakayokuwa tunauliza. Tafadhali jisikie huru kujibu vile unavyojisikia kujibu. Tuheshimu mawazo ya kila mshiriki. Kila utakachokisema hapa ni siri. Hatutataja jina lako nje ya chumba hiki/mahali hapa. Kama hutaki kujibu swali fulani hiyo ni sawa, kama unataka kuondoka wakati wowote hiyo ni sawa. Ila ninawasii sana tushiriki hadi mwisho wa majadiliano haya kwani mawazo yenu niya msingi sana katika utafiti huu.

Ninawaomba mturuhusu kurekodi/kuandika majadiliano ya mkutano.

Kujitambulisha (umri, elimu, kazi)

1. **Nini maoni yako kuhusu ushiriki wa wanaume katika uangalizi wa afya za wake/wenzi wao wakati wa ujauzito, kujifungua na baada ya kujifungua?**

Probes: Mwitikio wa wanaume kwenda kliniki na wake zao ukoje?
Wanaume wanawasaidia kazi wenzi wao/ wake zao wakati wa ujauzito na baada ya kujifungua?

2. **Je unafikiri ni muhimu wanaume kujadili na wenzi wao/wake zao kuhusu wanaume kwenda kliniki na wake zao wakati wa ujauzito’ kujifungua na baada ya kujifungua?**

Probes: Nini faida ya uamuzi huo kwa mwanamke, mtoto aliyeko tumboni na kwa mwanaume?
Ni kwa namna gani ushiriki wa mwanaume utaongeza ubora wa huduma za kliniki ya wajawazito, kujifungua na huduma baada ya kujifungua na kuongeza idadi ya wakina mama kutumia huduma hizi?
Kushiriki kwa wanaume kutaleta mabadiliko yoyote katika huduma ya kliniki ya wajawazito, huduma ya kujifungua na baada ya kujifungua?

3. **Je wanaume katika kijiji hiki/jamii hii wanawasindikikiza wake zao/wenzi wao katika kliniki ya wajawazito, kujifungua na kliniki baada ya kujifungua?**

Probe: Ni faida gani wanapata wanaume wakihudhuria kliniki pamoja na wenzi/wake zao?
Mama anapata faida gani?
Mtoto aliyetumboni na mtoto aliyezaliwa anapata faida gani?
Baba wa mtoto anapata faida gani?

4. **Ni sababu zipi zinawazuia wanaume kwenda klininiki na wake zao/wenza wao wakati wa ujauzito, kujifungua na baada ya kujifungua?**

Probe: Mila na desturi? Hali ya kiuchumi? Taarifa kuhusu kuhudhuria kliniki?
Masuala yanayohusiana na kituo cha huduma za afya?
Kukosa uelewa kuhusu kinachofanyika katika kituo cha kutolea huduma?
Mambo yanayohusiana na sera ya afya ya nchi?

5. **Je wanaume katika kijiji/jamii hii wanawasaidia wenzi wao/wake zao kazi za nyumbani katika kipindi cha ujauzito, kujifungua na baada ya kujifungua?**

Dodosa:Wanawapumzisha wenzi wao? Wanaosha vyombo? wanapika? Wanalea watoto? Wanachota maji? Wanaenda shamba? Wanafua nguo? Wanasafisha nyumba?

Asante sana kwa muda wenu na maelezo mliyonipa.

Unaswali lolote au maoni kuhusu haya tuliyojadili?

(Jibu maswali yatakayoulizwa na washukuru washiriki kabla ya kufunga kipindi)
