# Supplementary material for: Community perspectives: An exploration of potential barriers to men’s involvement in maternity care in a central Tanzanian community
Source: PLoS One. 2020 May 21;15(5):e0232939. doi: 10.1371/journal.pone.0232939 (PMC7241761; doi:10.1371/journal.pone.0232939)
Supplement: S3 Appendix — (DOC) [file pone.0232939.s003.doc]

**Consolidated criteria for reporting qualitative studies (COREQ): 32-item checklist**

| No Item | **Guide questions/description** |
| --- | --- |
| Domain 1: Research team and reflexivity | |
| **Personal Characteristics** |  |
| 1. **Interviewer/facilitator**   The first author conducted all the FGDs, the second author acted as an observer for the FGDs. The second author conducted IDIs and first author took notes and recorded the interviews. **( see page 9-10, under data collection section, line #226-232)** | Which author/s conducted the interview or focus group? |
| 1. **Credentials**   The first author was a PhD candidate and the second author was a PhD holder | What were the researcher’s credentials? E.g. PhD, MD |
| 1. **Occupation**   The first author was an assistant lecturer and the second author was a senior lecturer. | What was their occupation at the time of the study? |
| 1. **Gender**   Both researcher were female | Was the researcher male or female? |
| 1. **Experience and training**   The first researcher has an experience in public health. She has served in government institution as a clinician and a nutritionist in hospital setting for several years before joining the university service as an assistant lecturer in sociology of health and illnesses, human growth and development as well as human nutrition. The second researcher has an experience in applied anthropology and community development work. She has served in civil service as a community development worker, prior to joining the university service as a student advisor. She has worked in various universities within and outside the country in both academic and administrative positions and did multi-disciplinary research and published its outcome in both local and international journals. **(see page 13, line #311-312, under trustworthiness section** | What experience or training did the researcher have? |
| **Relationship with participants** |  |
| 1. **Relationship established**   Yes, first author stayed for more than 6 months prior to data collection to become familiar with the settings**(see page 12, line #296-302, under trustworthiness section)** | Was a relationship established prior to study commencement? |
| 1. **Participants’ knowledge of the interviewer**   The reason for doing the research was explained to participants prior to actual data collection **(page10 line#237-238, under data collection)** | What did the participants know about the researcher? e.g. personal goals, reasons for doing the  research |
| 1. **Interviewer characteristics**   All authors had experience in working with community welfare and they were both involved in data collection and analysis. This could have created author’s bias. However the use of member checking technique increased the trustworthiness of the finding. **(page 37, line#1051-1053** | What characteristics were reported about the interviewer/facilitator? e.g. Bias, assumptions,  reasons and interests in the research topic |
| **Domain 2: study design** | |
| **Theoretical framework** |  |
| 1. **Methodological orientation and Theory**   The study was guided by data-driven thematic analysis **(see page 11, data analysis section)** | What methodological orientation was stated to underpin the study? e.g. grounded theory,  discourse analysis, ethnography, phenomenology, content analysis |
| **Participant selection** |  |
| 1. **Sampling**   Participants were purposefully selected **(see page 8, under sampling procedure section)** | How were participants selected? e.g. purposive, convenience, consecutive, snowball |
| 1. **Method of approach**   Face to face for FGDs and Telephone for IDIs participants **(see page 9, under data collection section)** | How were participants approached? e.g. face-to-face, telephone, mail, email |
| 1. **Sample size**   Total of 246 participants for focus group discussions and 44 participants for in-depth interviews **(see page 9, under data collection section)** | How many participants were in the study? |
| 1. **Non-participation**   10 people (5 couples) refused to participate due to their busy schedule and 2 community leaders refused due to lack of time **(see page 9, under data collection section)** | How many people refused to participate or dropped out? Reasons? |
| **Setting** |  |
| 1. **Setting of data collection**   The FGDs took place in the village or ward offices or in the nearby classroom, depending on the convenience of participants. The IDIs took place in the hamlet or street leader’s offices or the health care provider’s offices or village health care worker’s offices. **(see page 10, under data collection section)** | Where was the data collected? e.g. home, clinic, workplace |
| 1. **Presence of non-participants**   No any other person was present apart from the participants and researchers. **(see page 10, under data collection section)** | Was anyone else present besides the participants and researchers? |
| 1. **Description of sample**   The participant’s age ranged from 18 to 70 years. The majority of the participants (77%) had completed primary education and most of them (70%) were engaged in agricultural activities. The data were collected from December 2016 to June 2017. **(see page 10, under data collection section)** | What are the important characteristics of the sample? e.g. demographic data, date |
| **Data collection** |  |
| 1. **Interview guide**   Prior to data collection the interview protocols were pretested and adjustments were made. The interview protocols consisted of questions and prompt to guide the sessions. **(see page 9, under data collection section)** | Were questions, prompts, guides provided by the authors? Was it pilot tested? |
| 1. **Repeat interviews**   Because the interviews were reordered, we did not repeat any interview | Were repeat interviews carried out? If yes, how many? |
| 1. **Audio/visual recording**   We used the audio to collect the data**. (see page 9, under data collection section)** | Did the research use audio or visual recording to collect the data? |
| 1. **Field notes**   Field notes were made during the interviews and focus group. **(see page 9, under data collection section)** | Were field notes made during and/or after the interview or focus group? |
| 1. **Duration**   Interviews lasted between 30 and 45 minutes and focus group discussion between 50 and 70 minutes. **(see page 10, under data collection section)** | What was the duration of the interviews or focus group? |
| 1. **Data saturation**   This was discussed before we decided not to continue with data collection. **(see page 9, under data collection section)** | Was data saturation discussed? |
| 1. **Transcripts returned**   Yes, the transcripts were returned to participants for comments. **(see page 10, under data collection section)** | Were transcripts returned to participants for comment and/or correction? |
| **Domain 3: analysis and findings** | |
| **Data analysis** |  |
| 1. **Number of data coders**   Two data coders coded the data. **(see page 11, data analysis section)** | How many data coders coded the data? |
| 1. **Description of the coding tree**   Yes, figure 1, presents this | Did authors provide a description of the coding tree? |
| 1. **Derivation of themes**   They were derived from the data. **(see page 11, data analysis section)** | Were themes identified in advance or derived from the data? |
| 1. **Software**   QSR NVivo 9 software developed by QSR International**(see page 11, data analysis section)** | What software, if applicable, was used to manage the data? |
| 1. **Participant checking**   Some of the participants provided feedback | Did participants provide feedback on the findings? |
| **Reporting** |  |
| 1. **Quotations presented**   Quotations from different participants were presented to illustrate the themes in order to converse its meaning to the reader and to add transparency and trustworthiness of the findings and interpretations of the data. Participant’s quotation was identified by status or position and number as shown in the result section of the manuscript**(see result section)** | Were participant quotations presented to illustrate the themes / findings? Was each  quotation identified? e.g. participant number |
| 1. **Data and findings consistent**   Yes, The findings were discussed to reflect the data presented. **(see result section)** | Was there consistency between the data presented and the findings? |
| 1. **Clarity of major themes**   Yes, The major themes were clearly presented in the findings **(see results section)** | Were major themes clearly presented in the findings? |
| 1. **Clarity of minor themes**   Yes , the minor theme “pregnancy outside wedlock” emerged and was discussed **(see discussion section, page 36, line 2028-1031)** | Is there a description of diverse cases or discussion of minor themes? |
