# Supplementary material for: Community perspectives: An exploration of potential barriers to men’s involvement in maternity care in a central Tanzanian community
Source: PLoS One. 2020 May 21;15(5):e0232939. doi: 10.1371/journal.pone.0232939 (PMC7241761; doi:10.1371/journal.pone.0232939)
Supplement: Attachment — Submitted filename: Response to Reviewers Comments.DOC [file pone.0232939.s004.DOC]

We thank the reviewer for the valuable comments which have greatly improved our manuscript. The table below presents a point by point response to Reviewer’s comments.

| **Section** | **Comments** | **Responses** | **Remarks** |
| --- | --- | --- | --- |
| Additional Editor Comments | | | |
| Abstract and Manuscript body | The objective as stated in the abstract is slightly different from the one in the manuscript body | The objective in the main manuscript has been revised to match with research title reflected in the abstract | See correction in page 7 line #173 to175 |
| Methods | Please explain more about data collection procedures in the manuscript. For instance, data collection tools are mentioned in the abstract but not in the main text. Include some of the information you have provided in the checklist you attached. | This section has been explained in details to include the important information that has been missing including; how were participants for the FGDs selected, inclusion and exclusion criteria, Who conducted interviews and FGDs, …. to mention the few. | Inserted in pg 12 line #242 to 292 |
| COREQ checklist | The checklist you provided is inadequate. Please include a 32-item COREQ checklist specifying the Item, Guide question, and the Page/section in your manuscript where this is reported. | The checklist has been revised to include all 32-items and the page/section where it is reported has been indicated in the manuscript | This part has been revised to improve clarity |
| Reviewer #1 | | | |
| Major revisions | The effect of men’s involvement on health outcomes for women and children is directly linked to men’s knowledge, education, attitudes and behaviours” (Introduction, lines 49-51). What, specifically, do the authors mean here? The phrase “directly linked to” is not clear to me. | The phrase “directly linked to” has been omitted and replaced with the phrase “associated with”  Also two sentences has been added to the improve clarity of the paragraph | See correction in page 4 line #93 to 94  Inserted in pg 4 line #94 to 97 |
|  | The phrase “demonstrations of maleness” (Introduction, lines 56-57) is unclear. | This has been rephrased for better clarity as follows:  “In such situations where there is disregard of women’s views and deprivation of women’s access to household resources, women’s and children’s health status is affected.” | See correction page 4 , line#102 to 105 |
|  | The authors engage with complex and nuance concepts in the manuscript, and the language they use does not adequately express their points.  I suggest that the authors clarify and narrow the aims and scope of the manuscript, in order to more deeply engage with their material. | The manuscript has been revised to streamline arguments and improve clarity |  |
| Minor essential revisions | In my opinion the authors are inaccurate when they state that studies investigating barriers to men’s involvement in maternity care are limited (Introduction, line 74). There is a substantial and rapidly growing body of work in this area. | Revisited the statement to mean… “However, studies investigating men’s involvement in maternity care and their barriers in Tanzania are limited” | The word in Tanzania has been inserted in pg 6, line #144 |
|  | Could the authors provide more detail on the specific gap in the literature that they have identified? | The section has been revised to clearly show the gap | See rephrasing in Pg 7 line#168 to 172 |
|  | The authors should also provide citations for some of the studies they mention that are focused on male involvement in PMTCT (Introduction, lines 72-73). | The following citations has been inserted [10,25,28,34,51] | Inserted in pg 6 line #143 to 144 |
|  | The study does not include the perspectives of single, unmarried, and first-time parents. The implications of this on study findings should be discussed as a limitation. | Single and unmarried parents were not part of our study population because we wanted to obtain information from couples who were living together. The first-time parents were included in the study as long as there were living together and had a child of age 2 or below years.  The implication of excluding single and unmarried parents has been included in the limitations section | Inserted in page 52, line #1466 to 1468 |
|  | What are the “distinctive characteristics” of the study sites (Study area, line 112)? | This has been addressed, the paragraph has been added to elaborate those distinctive characteristics as seen in the manuscript | Inserted in line# 193 to 201 in pg 8 |
|  | Additionally, qualitative sampling is typically designed to explore diversity, rather than to be representative, so the rationale for sampling diverse sites could be rephrased. | Revisited the statement to read “The districts were selected based to their diverse characteristics of each in relation to male involvement” | See correction in page 8 line #191 to 193 |
|  | The themes ‘Women’s desire to have their partners in the antenatal clinic’, ‘Health care system barriers’, and ‘Location of the health care facilities’ would benefit from the addition of more illustrative quotes, to flesh out key findings and fully articulate the theme. | The theme “Women’s desire to have their partners in the antenatal clinic” has been renamed as “Benefits of women men’s involvement in maternity health care services”  More illustrative quotes have been added as seen in the manuscript  The theme “Location of the health care facilities” has been renamed as “Distance to the health care facilities”  More illustrative quotes have been added as seen in the manuscript | Inserted in page 20, line# 535  Page 21, line# 559 to 568  See page 32, line #904  Page 33, line#956 to 960 |
| Discretionary, but recommended, revisions | The authors may wish to include a visual summary of their themes, to replace or complement the text summary in the first paragraph of the Results section. | A visual summary of themes has been presented as per reviewer’s comment | See Fig 1. |
|  | The attributions for illustrative quotes (e.g. “FGD(2)-male participant Msalato_Dodoma municipal”) are distracting and could be formatted differently. | The attributions for all illustrative quotes has been formatted sequentially from 1-32 as seen in the manuscript | See the results section pg 15 to 36, line #363 to 1054 |
| Reviewer #2 | | | |
| General comments | There are many editorial errors, unclear and or sensitive and inconsistently used words. | The whole documents has been revised and editorial work has been done as seen in the manuscript |  |
|  | The authors need to recast the title to make it short and clear. Delete unnecessary repetition of words in the title. | The title has been revised and it now reads as “Community perspectives: an exploration of potential barriers to men’s involvement in maternity care in a central Tanzanian community  ” | See corrected title page |
| Abstract | Line 26- need to be clear if they used content or thematic approach in data analysis. In the paper, they talk about thematic rather than content analysis. | The study employed thematic approach during data analysis and the abstract has been revised accordingly | See corrected abstract |
|  | Line 44 – chores instead of chore | The whole section has been revised for better clarity. | See revision in page 2, line# 40 to 45 |
| Introduction | The concept male involvement should be comprehensively defined not just focusing on clinic attendance. Similarly, the discussion on male involvement should include psychological, medical, socio-cultural etc. The authors need to include empirical evidence on the benefits and potential harms of male involvement in maternal health care | More explanation has been added to improve clarity as seen in the introduction section.  For scope and manageability the study focused on two aspects namely; attendance in ANC and workload support during maternity period | See revision in page 3 to 6, line# 66 to 136 |
|  | There is need to clearly indicate the knowledge gap and how the study advances existing knowledge. | The section has been revised to clearly show the gap | Inserted in Pg 7 line#168 to 172 |
|  | Line 49-51, recast this sentence. Similarly, line 56 -58. | The phrase “directly linked to” has been replaced with “associated with” and sentence had been recast. | See correction in pg 4, line#93 to 97 |
|  | Line 61, add word use or uptake after contraceptive. | The word ”use” has been inserted after the word “contraceptive” | Inserted in Pg 4 line#108 |
|  | Line 62, delete word maternal after increase | The word “maternal” has been removed | See correction in Pg5 line#109, |
|  | Line 66 – there is need also to highlight potential harms of male involvement in maternal health care. | This has been addressed | See addition in pg6, line# 127 to 136 |
|  | Line 69-73 – recast this sentence. | The sentence has been recast | See correction page 6, line# 138 to 145 |
|  | Line 72 – the concept maternity care needs to be clearly defined. | This has been defined as per reviewer’s comments | Inserted in Pg 3line#67 to 74 |
| Materials and methods | More information is needed on methods and data collection. For example, how were participants for the FGDs selected? What were the inclusion and exclusion criteria? Who conducted interviews and FGDs? Where were interviews and FGDs conducted? How were the number of interviews and FGDs determined - how saturation point was reached? How FGDs were organized? How quality was maintained during data collection? How was the Nvivo 9 software used in the data analysis. | The whole section has been revised to improve clarity of the manuscript | Inserted in pg 10 to 12, line #242 to 292 |
|  | More clarity is needed on the data analysis. The qualitative analysis part is rather simplistic and unclear. For example, who coded the data? How codes were determined? How was the coding done? Which themes emerged and how these themes were generated? | The whole section has been revised to improve clarity on data analysis as indicated in the manuscript | See correction in pg 12 to 13, line #297 to 331 |
|  | Line 111 – 112 not clear what the authors mean. | The sentence has been rephrased to improve clarity | See correction in pg 8, line # 191 to 201 |
|  | Line 114 – 120 recast the paragraph, similarly, line 122 – 136. | The paragraph has been revised, unclear statements have been omitted as seen in the manuscript | Omitted line #191 to 201 in page 8 to 9 |
| Results | This section is unnecessarily too long. There is need to reduce the number of quotes and only use them when necessary. | The number of quotes have been reduced and used only when necessary as seen in the manuscript | See the results section page 15 to 36, line #363 to 1054 |
|  | There is no table that shows the themes and codes identified under each theme and makes it difficult to comment on how the codes are interpreted and how similar codes are categorized in to the same theme. However, some of the themes need to be merged to avoid repetition. For example, there are many repetition of what is discussed in the workload support and cultural barriers to male involvement. | Thematic figure showing codes, themes description and analytical themes has been prepared  Repetitive codes/themes have been merged | See fig 1. |
|  | While the authors indicate that they conducted 32 FGDs, throughout the results section all quotes only refer to FGD 1 and FGD 2. There is also confusion in many quotes. Sometimes FGD 1 indicate female participants and in other places male participants. Similarly, FGD 2 in some places they refer female participants while in other places male participants. | The illustrative quotes from FGDs have been sequentially presented from 1-32 as seen in the manuscript | See the results section page 15 to 36, line #363 to 1054 |
|  | Line 166 – 175 – need to summarize this paragraph | The whole section has been revised | See revision in page 15 to 16, line# 363 to 386 |
|  | Line 315 – 317 – this theme needs to be expanded. It would also be good to get perceptions of men on this issue. | Revisited the theme to mean “Benefits of men’s involvement in maternity health care services**”** The theme has expanded and perceptions of men has been included | See revision in page 20 to21, line# 535 to 568 |
|  | Line 370 – 380 – the quote is extremely long | The illustrative quote has been shortened | See revision in page 23, line#623 to 630 |
|  | Line 441 – 461- this is different from pregnancy outside marriage. | This section has been omitted | See page 27 to 28, line#775 to 795 |
|  | Line 493 – 513 – The description of this theme is not focused. Many issues are mixed together and thus difficult for the reader to understand. | The theme has been renamed and it is now “Precarious family and economic situations” and only relevant illustrative quotes have been maintained and its description is now focused | See revision in page 28, line#833 to 856 |
|  | Line 515 – this theme could better be named as lack of knowledge. | The theme has been Revised to mean **“**Lack of knowledge and inadequate information**”** | See revision in pg 29, line# 857 |
|  | Line 528 – replace word nevertheless with an appropriate word. | The word has been removed | See deletion page 30, line 886 |
|  | Line 550 – make the theme short, it reads like a sentence rather than theme | The theme name has been shorten to “Long waiting time” | See revision in page 31, line 908 |
|  | Line 582 – 586 – this theme needs to be expanded. Why women are able to attend health facilities despite the long distance? why not men? Are women in these communities physically stronger than men? | The theme has been expanded to clear the doubt | See correction in page 32 to 33, line#940 to 964 |
|  | Line 607 –619- small consultation room is not discussed in the text. Only privacy in the labour room is discussed. | The section has been revised for more clarity and relevant illustrative quote has been added | See correction in page 34, line#922 to 1011 |
|  | Line 621 – 640 – This theme is not clear. In Tanzania, maternal and child health services are not charged. How then do shortage of medical supplies hinder men’s participation in maternal and child health services? | This has been addressed clearly in the manuscript | Seen revision in page 34 to 36, line#1013 to 1054 |
| Discussion | Overall, the discussion is weak and unnecessarily long. The discussion part is not different from the Finding. No new arguments that lead to conclusion. There is no critical analysis and reflection of the findings. Repeatedly, findings are compared with studies conducted in other countries. The authors need to discuss implications of the findings in Tanzania context. | The whole discussion section has been revised | See the discussion section |
|  | Line 653 – 658 – These concepts should have been introduced in the results section. Don’t introduce new concepts in the discussion. | The concepts are in the results section | See the results section page 23 to 24, line#648 to 683 |
|  | Pg 21- how are the two paragraphs different? Both seem to refer to cultural gender roles in the community. | The whole section has been revised including these paragraphs | See the discussion section |
|  | Line 682 – 683 – Which study area are you referring to? | It was refereeing to central part of Tanzania. However this section has been revised | See revision in page 8, line# 189 to 201 |
|  | Line 683 – 686 – no need of repeating methods | This section has been revised | See the discussion section |
|  | Line 704 – 708 – This was not discussed in the results section. Don’t introduce new findings in the discussion. | This section has also been revised | See the revision in the discussion section |
|  | Line 709 – 711 – The authors need to explain why women were able to attend despite long distance? | The statement has been qualified in the discussion section | See the discussion section |
|  | Line 711 – 715 – This strategy already ended in 2017 (2007 -2017). How can implementation then be accelerated? | The recommendation has been recast | See the revision in the discussion section page 39, line#1143 |
|  | Line 770 – 771 – No findings on the consultation rooms was reported in the results section. | The section has been revised and the relevant illustrative quote has been added | See revision in page 34, line#922 to 1011 |
|  | Line 779 – 786 Need to refer to the correct name of the Ministry. This Ministry has changed since 4 years ago. | The ministry name has been corrected as “Ministry of Health, Community Development, Gender and the Elderly (MoHCDGE)” | Seen correction in page 39, line#1143 |
|  | Line 788 – 791 This was not discussed in the results section. Don’t introduce new findings in the discussion. | The section has been revised | See the discussion section |
| Strengths and weaknesses | How can findings of a qualitative study conducted in 16 villages be generalized for the entire country? The authors need to understand the purpose of conducting qualitative studies and why they differ from quantitative studies. | The strengths and weaknesses section has been revised and relevant information has been added | See revision in page 52, line#1453 to 1475 |
| Conclusions and recommendations | There is no conclusion; the current conclusion is rather a summary of findings. The authors need to provide conclusions of the paper. Recommendations are generally weak. The authors should provide recommendations which are aligned to the findings of the study. | The conclusion and recommendation section has been revised, reflecting the theoretical and practical implication of the findings | See revision in page 53 to54, line#1479 to 1514 |
