# Supplementary material for: Community perspectives: An exploration of potential barriers to men’s involvement in maternity care in a central Tanzanian community
Source: PLoS One. 2020 May 21;15(5):e0232939. doi: 10.1371/journal.pone.0232939 (PMC7241761; doi:10.1371/journal.pone.0232939)
Supplement: Attachment — Submitted filename: Response to ReviewersComments.docx [file pone.0232939.s005.docx]

We thank the reviewers and academic editor for the valuable comments that have greatly improved our manuscript. The table below presents a point-by-point response to Reviewer’s and Academic Editor’s comments.

| **Comments** | **Responses** |
| --- | --- |
| Editor’s Comments | |
| I notice that you sometimes use the present tense in the results section (for example line 423). Can you please revise this section accordingly? | The section has been revised and is now in the past tense as seen in the manuscript |
| You can also use this opportunity to proofread the entire manuscript and correct any grammatical errors. For instance, you talk of "Men's attendance to maternity health services". I think it is correct to say "Men's attendance of maternity healthcare services..." | Editorial work has been done in the entire document as seen in the manuscript. However, we would prefer to use the word “at” instead of “of”. We think it is correct to say “Men’s attendance at maternity health care services” instead of “Men’s attendance of maternity health care services”. The correct grammar for attendance is “attendance at” and can’t get any other option |
